# Regional expression of the *MAPT* gene is associated with loss of hubs in brain networks and cognitive impairment in Parkinson disease and progressive supranuclear palsy

**DOI:** 10.1016/j.neurobiolaging.2016.09.001

**Published:** 2016-12

**Authors:** Timothy Rittman, Mikail Rubinov, Petra E. Vértes, Ameera X. Patel, Cedric E. Ginestet, Boyd C.P. Ghosh, Roger A. Barker, Maria Grazia Spillantini, Edward T. Bullmore, James B. Rowe

**Affiliations:** a Department of Clinical Neurosciences, University of Cambridge, Herchel Smith Building for Brain and Mind Sciences, Cambridge, UK; b Department of Psychology, Behavioural and Clinical Neuroscience Institute, University of Cambridge, Cambridge, UK; c Department of Psychiatry, Herchel Smith Building for Brain and Mind Sciences, Cambridge, UK; d Churchill College, University of Cambridge, Cambridge, UK; e Department of Biostatistics, Institute of Psychiatry Psychology and Neuroscience, King's College London, London, UK; f Wessex Neurological Centre, University Hospital Southampton, Southampton, UK; g John van Geest Centre for Brain Repair, University of Cambridge, Cambridge, UK; h Medical Research Council Cognition and Brain Sciences Unit, Cambridge, UK

**Keywords:** Tau, Progressive supranuclear palsy, Parkinson's disease, Gene expression, Functional connectivity, Brain imaging

## Abstract

Abnormalities of tau protein are central to the pathogenesis of progressive supranuclear palsy, whereas haplotype variation of the tau gene *MAPT* influences the risk of Parkinson disease and Parkinson's disease dementia. We assessed whether regional *MAPT* expression might be associated with selective vulnerability of global brain networks to neurodegenerative pathology. Using task-free functional magnetic resonance imaging in progressive supranuclear palsy, Parkinson disease, and healthy subjects (n = 128), we examined functional brain networks and measured the connection strength between 471 gray matter regions. We obtained *MAPT* and *SNCA* microarray expression data in healthy subjects from the Allen brain atlas. Regional connectivity varied according to the normal expression of *MAPT*. The regional expression of *MAPT* correlated with the proportionate loss of regional connectivity in Parkinson's disease. Executive cognition was impaired in proportion to the loss of hub connectivity. These effects were not seen with *SNCA*, suggesting that alpha-synuclein pathology is not mediated through global network properties. The results establish a link between regional *MAPT* expression and selective vulnerability of functional brain networks to neurodegeneration.

## 1 Introduction

Several neurodegenerative diseases are associated with the hyperphosphorylation, misfolding, and aggregation of the microtubule-associated protein tau, including progressive supranuclear palsy (PSP), frontotemporal dementia, and Alzheimer's disease. Genetic variations in the tau gene *MAPT* also increase the risk of PSP, frontotemporal dementia, Parkinson disease (PD) (Martin et al., 2001, Satake et al., 2009, Simón-Sánchez et al., 2009), and dementia in PD (Goris et al., 2007). Each of these disorders is associated with selective vulnerability of distinct brain regions within distributed functional brain networks (Zhou et al., 2012). Although there are suggestions that tau haplotype may contribute (Winder-Rhodes et al., 2015) to its association with these diseases, the mechanisms behind this susceptibility remain unclear.

The misfolded and hyperphosphorylated tau protein may provide a pathogenic template for the propagation and spread of tau pathology. For example, in transgenic murine models, it has been shown that tau can propagate via trans-synaptic spread (Ahmed et al., 2014, Liu et al., 2012), which promotes the transformation of normally expressed tau to pathogenic species (Clavaguera et al., 2013). Here, we propose that high levels of the intrinsic expression of tau in humans may enhance this templating process, thereby accelerating the pathogenesis of neurodegenerative disorders associated with tau and *MAPT*, by promoting the genesis and spread of the abnormal tau species. We predicted that brain regions with a higher expression of tau in healthy adulthood are more susceptible to a loss of function in the human diseases. We predict this would be true in neurodegenerative syndromes associated with tau pathology such as PSP, as well as other diseases associated with genetic variation of *MAPT* such as PD. To test this hypothesis, we examined the regional expression of *MAPT* in a cohort of healthy subjects from the Allen Brain atlas.

We contrasted the effect in *MAPT* with *SNCA*, the gene encoding alpha-synuclein, which is associated with the pathology of PD but not PSP. Alpha-synuclein neuropathology in PD is localized to brainstem nuclei and the basal ganglia in early PD (Braak et al., 2003). In our population of PD subjects without dementia, we would therefore expect *SNCA* expression to have a limited effect on global network measures relevant to cognition.

We used functional brain networks to look for an association between genetic and clinical descriptions, both in a healthy older cohort and in neurodegeneration (Hawrylycz et al., 2015, Meyer-Lindenberg and Weinberger, 2006, Richiardi et al., 2015). The analysis of the organization of large scale functional brain networks in health and neuropsychiatric disease exploits the synchronization of activity across neuronal populations in functionally and anatomically defined networks (Bullmore and Sporns, 2009). Within these distributed networks, highly connected hub regions enhance network efficiency by connecting topologically remote regions (Power et al., 2013, Sporns et al., 2007). However, highly connected “hub” regions may be particularly susceptible to neurodegeneration in Alzheimer's disease (Buckner et al., 2009) and other brain disorders (Crossley et al., 2014).

We therefore started by examining the association between functional connection strength in health and gene expression, then went on to associate connection strength and gene expression with the proportional loss of connection strength in PSP. We focused on hub regions to explore the link between functional connection strength and cognition in PD and PSP.

## 2 Methods

### 2.1 Subjects

A total of 146 subjects (60 age-matched controls, 30 PD without dementia Mini-Mental State Examination >26/30, 56 PSP) were recruited with written informed consent and approval from the local ethics committee. Of these, 16 were excluded after functional magnetic resonance imaging (fMRI) image preprocessing revealed a focal lesion, significant white matter hyperintensities, or motion dependent confounds on connectivity measures. In the remaining 128 participants (53 controls, 30 PD, 45 PSP), there were no group differences in age, gender, handedness or years of education; as expected, cognition was more impaired in PD and PSP groups using the MMSE (Table 1). For motor function, we report both the Unified Parkinson's Disease Rating Scale, and for motor and nonmotor symptoms in the PSP group, the PSP Rating Scale (PSPRS) (Golbe and Ohman-Strickland, 2007). Verbal fluency testing required subjects to generate words beginning with the letter “p” (letter fluency) or animals (category fluency), each for 1 minute. These 2 scores were added together to produce a combined verbal fluency score.

Microarray data from 6 postmortem brain samples were obtained from the Allen Institute at http://human.brain-map.org (H0351.1009, H0351.1016, H0351.1015, H0351.2002, H0351.1012, and H0351.2001), mean age 45.5 (range 24–57), all male, 4 right-handed, 1 left-handed, and 1 ambidextrous.

### 2.2 Image preprocessing

Imaging was performed at the Wolfson Brain Imaging Centre, on a 3T Tim TRIO Siemens MRI scanner. Medication was not altered before scanning. Task-free echo-planar fMRI was acquired for a minimum of 145 volumes with subjects instructed to keep their eyes open (repetition time [TR] = 2 seconds, echo time [TE] = 30 ms, flip angle = 78°, voxel size = 3 × 3 × 3.75 mm) and structural Magnetization-Prepared Rapid Acquisition with Gradient Echo scanning in the same session (TR = 2.3 seconds, TE = 2.98, flip angle = 9°, voxel size = 1 mm isotropic).

Preprocessing of structural scans involved optimized skull-stripping for atrophied brains (Acosta-Cabronero et al., 2008) using affine transformation followed by nonlinear warping using the FSL FNIRT tool to a group-specific template to ameliorate the challenge of warping atrophied brains before nonlinear transformation to Montreal Neuroscience Institute (MNI) standard space. Functional image preprocessing was tailored to minimize the effect of motion, which can influence the correlation between distance and connectivity (Patel et al., 2014), and atrophy in neurodegenerative disease. The pipeline used a customized version of the fMRI signal processing toolbox (www.brainwavelet.org) and included discarding the first 5 volumes, slice-timing correction, coregistration to the Magnetization-Prepared Rapid Acquisition with Gradient Echo image, motion correction, regression of motion derivatives, white matter and CSF signal, local median despiking, high-pass band filtering of 0.01 Hz, and normalization to MNI standard space using parameters obtained from normalization of the structural image (performed in the described order). Quality control included (1) visual inspection at each preprocessing stage; and (2) measuring the influence of motion on correlation assessed using delta-BOLD and framewise displacement correlations, in a modified protocol from Power et al. (2011) and Satterthwaite et al. (2012). More specifically, subjects were excluded if there was greater than 5-mm framewise displacement between any 2 TR intervals and if there was a significant correlation between motion and the change in BOLD signal measured by wavelet correlation before and after a “scrubbing” procedure (delta-BOLD).

### 2.3 Network analysis

A parcellation scheme divided gray matter of the MNI standard space template in to 500 nonoverlapping regions of equal size, using centroidal Voronoi tessellation (Du et al., 1999). After the random selection of 500 centroids, each region was constructed by assigning the remaining voxels to one of the different centroids. This regional allocation was performed by minimizing the Euclidean distance between each voxel and the set of centroids, thereby producing a 3-dimensional Voronoi tessellation. When ties occurred, these were broken using random allocation. Consequently, each region was composed of contiguous voxels with a mean size of 364.7 voxels (sd 176.7).

Of the 500 regions, 29 were insufficiently covered by the fMRI scans in some subjects, leaving 471 brain regions for further analysis. Individual subjects' association matrices were constructed by extracting a time series from each parcel and performing a wavelet decomposition using a maximal overlap discrete wavelet transform and a Daubechie wavelet filter using the brainwaver package in R (http://cran.r-project.org/web/packages/brainwaver). The second band of 4 corresponding to a frequency range of 0.0675–0.125 Hz was selected for further analysis (Achard et al., 2006). Graphs were constructed from association matrices without thresholding (negative weights retained) and based on the Fisher z-transforms of the correlation coefficients, and after wavelet decompositions of the fMRI time series. Network analysis was carried out in python 2.7 using the Maybrain package in python (https://github.com/rittman/maybrain) drawing on the networkx package (1.8.1, http://networkx.github.io).

For comparison with microarray data, a mean association matrix was constructed for each diagnostic group. This was generated by calculating the mean connection strength of each brain region from all subjects within a diagnostic group.

### 2.4 Hub definition and connectivity loss

Verbal fluency scores were correlated with the mean connection strength of the most highly connected “hub” regions. In the context of this paper, a hub was defined as brain region whose connection strength (the sum of all its connections) was above a threshold of 1.5 standard deviations of the mean connection strengths of all regions, based on the association matrix in control subjects (see Supplementary Fig. 1 demonstrating the location of hubs).

The proportion of connectivity loss between the control group and PD or PSP group was calculated as the difference in connection strength between groups for each brain region normalized by the connectivity in the control group (Achard et al., 2012). Note that proportionate, rather than absolute, loss of hub connectivity is used, as hubs have by definition higher absolute connectivity, whereas our hypotheses refer to the proportionate vulnerability of hubs to the progression of neuropathology.

### 2.5 Microarray analysis

Microarray data available from the Allen brain atlas was generated using an Agilent 8 × 60 K array, custom-designed by Beckman Coulter Genomics. From this data, we identified 4 probes for the *MAPT* gene (A_23_P207699, A_24_P224488, A_32_P143793, and CUST_449_PL1416408490) and 2 probes for the *SNCA* gene (A_32_P109653 and A_23_29,939). The specificity of each probe was reassessed using nucleotide BLAST (http://blast.ncbi.nlm.nih.gov/Blast.cgi) to identify genes and translated nucleotide sequences associated with each probe's sequence. Three of the 4 *MAPT* probes met our criteria: 100% homology with an area within the *MAPT* gene and associated transcripts and no other regions of homology, with very small E values to suggest good sensitivity. These 3 probes matched regions in the *MAPT* gene, A_23_P207699 and A_24_P224488 homologous with the gene sequence to target complementary DNA and A_32_P143793 with the anti-sense sequence targeting complementary RNA. *MAPT* has 17 exons and is alternately spliced into 6 isoforms, with 2 main groups differentiated by 3 or 4 repeats of the binding domain as a result of alternate splicing of exon 10. All probes recognized 3 and 4 repeat tau. The fourth probe (CUST_449_PL1416408490) showed only 88% homology with *MAPT* and 80% homology with a region on chromosome 18 and was discarded. Two probes in the Allen brain atlas data labeled as targeting *SNCA* (A_32_P109653 and A_23_29939) had 100% homology with regions in the *SNCA* gene with small E values. Both probes were homologous with complementary DNA. Further detail on the microarray analysis methodology is available from the Allen Institute (www.brain-map.org).

### 2.6 Comparison between microarray and functional network data

There were 3 challenges in identifying homologous regions between the genetic and imaging data: (1) only 2 Allen subjects have data from both hemispheres; (2) regions in the Allen data set are not evenly spaced; and (3) the number and precise location of regions differ between Allen subjects. To address the challenge of missing data from the right hemisphere in 4 Allen subjects, the remaining 2 subjects were used to correlate microarray expression data between the hemispheres. We used the remaining 2 subjects with whole brain data to assess whether it was reasonable to use the single hemisphere data from the other 4 subjects to represent whole brain. For each brain region, we calculated the Pearson correlation coefficient (Fisher's r-to-z transformed to enable statistical comparison) between the expression values of each hemisphere for 29,131 probes associated with an identified gene. Data for the null hypothesis were generated by permuting interhemispheric brain regions for 10,000 random region pairs. There was a significant difference between observed and permuted data for both subjects; subject A mean z = 0.46, *p* < 0.00001, subject B mean z = 0.40, *p* < 0.00001. We therefore analyzed *MAPT* and *SNCA* data under the assumption of symmetry of gene expression.

To address the second challenge of different spatial distributions between imaging and genetic data, each imaging region was associated with the nearest brain region from all Allen subjects combined. Finally, each Allen brain region's anatomical label was used to identify comparable regions across the 6 Allen subjects, in standard anatomical space. Each of the 471 imaging regions was then paired with an anatomically labeled region of the Allen atlas (see Supplementary Fig. 2 demonstrating regions of the Allen brain atlas included in the final analysis). For each region, the gene expression value was calculated as the mean of all samples falling in to the appropriate brain regions from all 6 Allen subjects.

### 2.7 Statistical analysis

Statistical analysis was performed in R (Version 3.0.1 http://cran.r-project.org). Groupwise differences in node degree were tested using a *t* test with *p*-values obtained using permutation testing (5000 permutations) and corrected for multiple comparisons using the false discovery rate (Benjamini and Yekutieli, 2001). Correlation between *MAPT* or *SNCA* expression and node degree used the Pearson correlation coefficient and *p*-values estimated from permutation testing. To assess the relationship between verbal fluency and the proportional loss of connection strength in PD and PSP, a 1-way analysis of variance was performed assessing the main effect of connection strength on verbal fluency.

## 3 Results

**Fig. 1:**
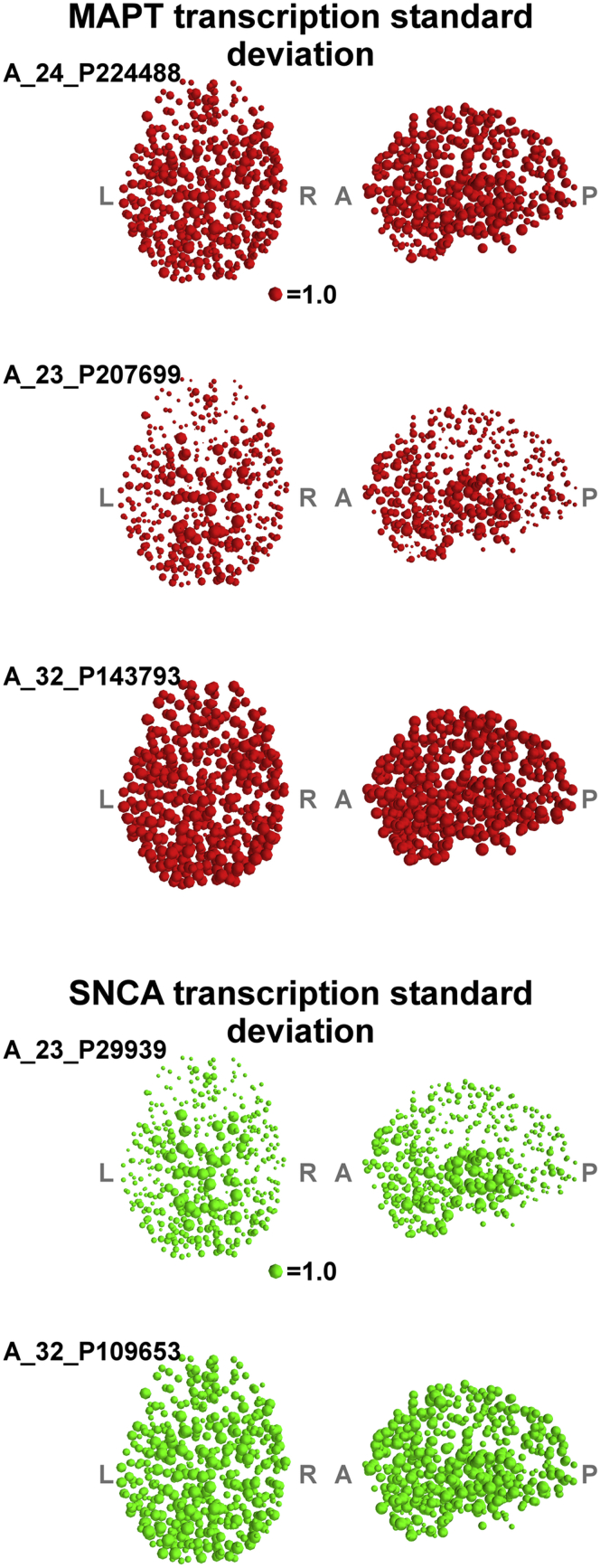
The figure demonstrates the variability in microarray expression data between the 6 Allen brain atlas subjects; the size of each sphere is scaled by the standard deviation of gene expression in the region. This was measured by normalizing the expression values within each subject to a mean of 0 and standard deviation of 1, then assessing the interindividual standard deviation at each brain region. These results confirm an acceptable variability between subjects, with less variability in cortical compared with subcortical regions. There were no regions with an unacceptably high variability between subjects.

**Fig. 2:**
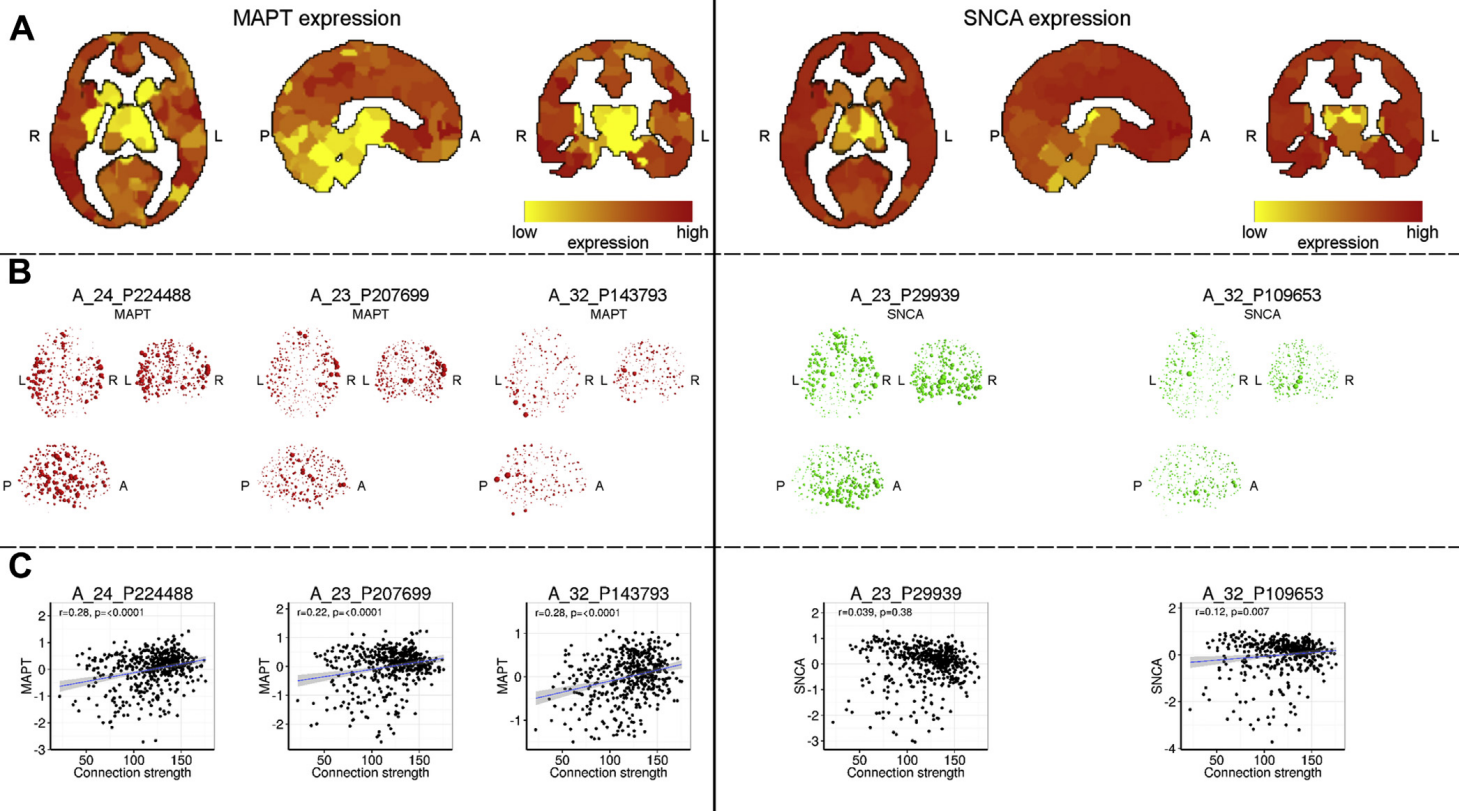
The relationship between functional connection strength in control subjects and expression of *MAPT* and *SNCA* genes. The connection strength was measured in control subjects as the mean r-to-z transformed wavelet cross-correlation between the task-free functional magnetic resonance imaging activity of a region and all other regions. Genetic microarray expression data were obtained from the Allen brain database by matching regions in the Allen brain to brain regions imaged and aggregating expression values for gene probes across subjects and normalized within each subject. (A) Shows the distribution of expression values for *MAPT* and *SNCA* aggregated across microarray probes using an arbitrary scaling. In both genes, the cortex has greater gene expression than the cerebellum and subcortical regions. In *MAPT*, the posterior cortical regions including the precuneus and posterior temporal lobe regions demonstrate greatest gene expression. (B) Shows glass brain projections of expression values for each probe individually. (C) Shows significant correlations between the regional distribution of functional connection strength and the expression of *MAPT*, a correlation that remained when values from the 3 probes were aggregated (r = 0.29, *p* < 0.00001 corrected). One of the *SNCA* probes weakly correlated with connection strength, although this did not survive when combining both *SNCA* probes (r = 0.08, *p* > 0.1 corrected).

We measured functional connectivity among 471 of 500 gray matter regions during task-free (or “resting-state”) fMRI in 128 subjects: 30 people with PD, 45 people with PSP, and 53 age- and sex-matched healthy controls. Our principal measure of connectivity was the sum of connection weights to all other regions (hereafter connection strength), derived from the association matrix of z-transformed wavelet correlations. To assess the relationship between functional connection strength and gene expression, we extracted the *MAPT* expression for each of 3 *MAPT* probes and 2 *SNCA* probes in 6 subjects from the Allen Brain Atlas. Fig. 1 illustrates the intersubject variation (i.e., standard deviation) in gene expression for each probe. The expression values were normalized for each probe within each subject: the mean expression value for each probe was zero, and the standard deviation was 1 for each probe (across all regions). Fig. 2A and B illustrate the regional differences in the magnitude of *MAPT* and *SNCA* gene expression.

There were positive correlations between *MAPT* expression and the connection strength in control subjects when values from all 3 *MAPT* probes were combined (r = 0.29, *p* ≤ 0.00001) and when examined separately (Fig. 2C). The expression of *SNCA* did not correlate with connection strength when combining data across both probes (r = 0.08, *p* > 0.1), although the expression values of 1 probe correlated weakly with connection strength (A 32 P109653, r = 0.12, *p* = 0.036, see Fig. 2). Next, we related these effects to PD and PSP.

**Fig. 3:**
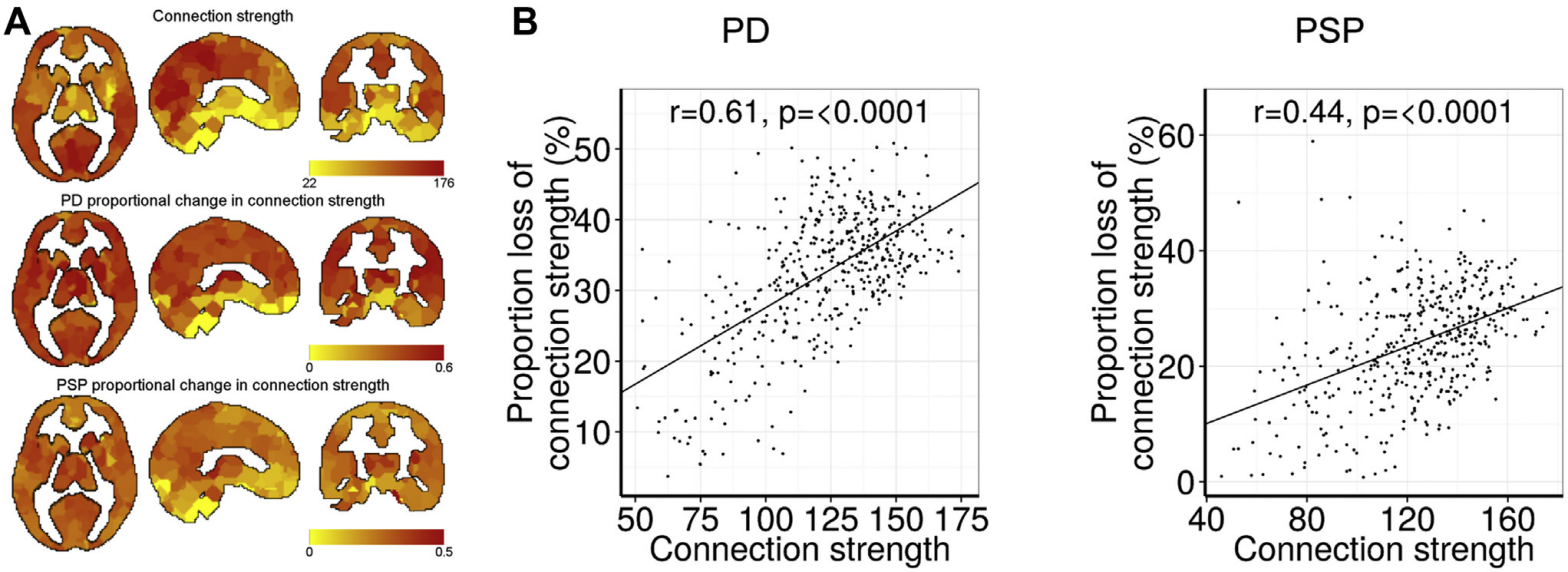
The proportionate (not absolute) loss of connectivity strength in PSP and PD subjects across 471 regions. Functional connection strength in health predicts the proportional loss of connection strength in both PSP and PD, supporting the hypothesis that more connected regions (including hubs) are particularly susceptible to neurodegeneration. (A) Shows the proportional loss of connection strength in disease by region. (B) Shows the significant relationships between the connection strength in control subjects and proportional change of connection strength in disease groups. Abbreviations: PD, Parkinson disease; PSP, progressive supranuclear palsy.

We assessed whether regions of high connection strength were susceptible to the effect of neurodegeneration in PD and PSP by measuring the proportional loss of connection strength across each of the 471 brain regions (Fig. 3). It is expected that regions with more connections would lose more of these in absolute terms, but the hypothesis of selective vulnerability posits that well-connected regions lose a disproportionate number of connections; therefore, our analysis examined the proportional loss of connection strength. Brain regions with greater connection strength in controls lost the largest proportion of connection strength in PD (r = 0.61, *p* < 0.00001 corrected) and PSP (r = 0.44, *p* < 0.00001). Brain regions that lost the greatest proportion of connection strength also had greater expression of *MAPT* in PD (r = 0.16, *p* = 0.0014, corrected for multiple comparisons across 2 genes using a Bonferroni correction) but not in PSP (r = 0.06, *p* > 0.1), and there were no significant associations with *SNCA* (PD: r = 0.02, *p* > 0.1, PSP: r = −0.08, *p* > 0.1).

**Fig. 4:**
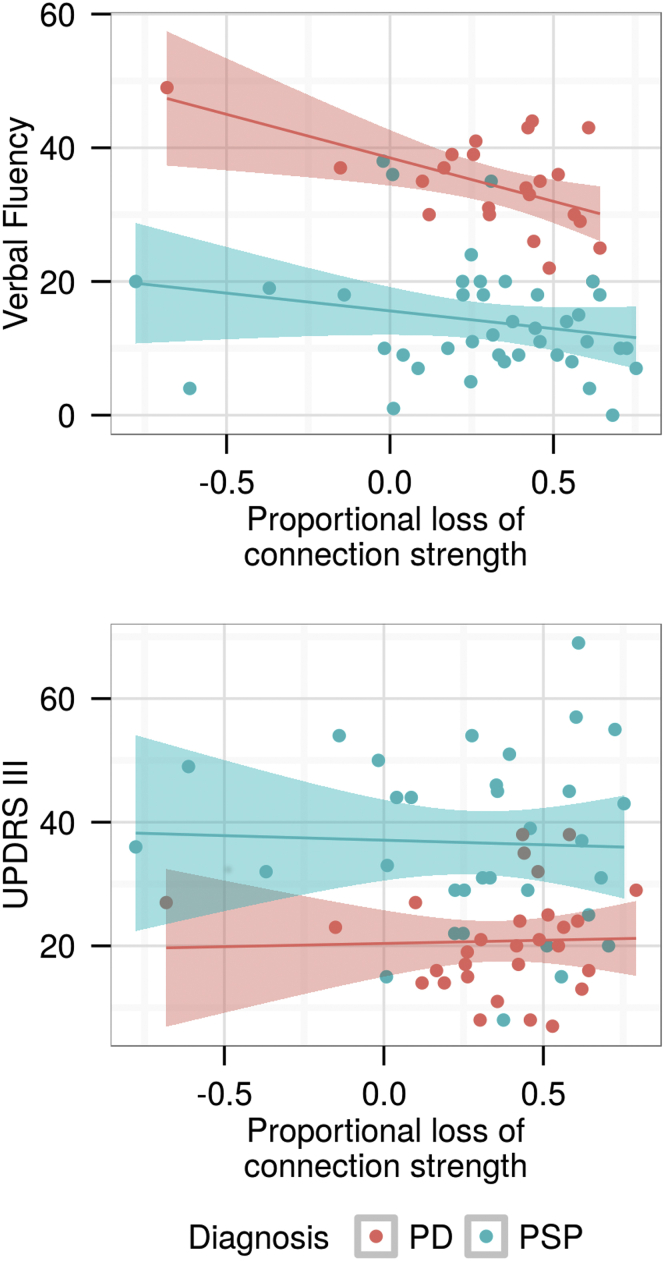
The connection strength in highly connected hub brain regions correlates with verbal fluency in PD and PSP (F = 6.1, df = 1, *p* = 0.02; post hoc *t* tests PD: r = 0.52, df = 21, *p* = 0.01; PSP: r = 0.21, df = 38, *p* > 0.1), but there was no correlation between hub connection strength and motor function measured using UPDRS (F = 0.02, df = 1, *p* > 0.1; post hoc *t* tests, PD: r = −0.035, *p* = 0.9; PSP r = 0.038, *p* = 0.8). Abbreviations: PD, Parkinson disease; PSP, progressive supranuclear palsy; UPDRS, Unified Parkinson's Disease Rating Scale.

Finally, we examined the relevance of connectivity changes to cognitive performance. Although PSP and PD have distinct clinical phenotypes, both diseases are associated with early impairment of executive function (Williams-Gray et al., 2007), which can be indexed by verbal fluency (Burrell et al., 2014). We identified the most highly connected “hub” brain regions (as previously mentioned) in control subjects and used the mean connection strength across hubs, applying analysis of covariance to account for the difference in distribution of verbal fluency scores of each group (Fig. 4). The mean verbal fluency scores were controls 40.3 (standard deviation 10.4), PD 34.3 (7.3), and PSP 14.1 (8.6). The connection strength of hub regions positively covaried with verbal fluency in both patient groups, that is, greater connection strength was associated with greater verbal fluency (F = 6.1, df = 1, *p* = 0.02). There was no interaction between this covariance and diagnosis (F = 1.3, df = 1, *p* > 0.1), although post hoc correlations demonstrated that verbal fluency declined with the loss of hub connectivity in PD (PD: r = 0.52, df = 21, *p* = 0.01; PSP: r = 0.21, df = 38, *p* > 0.1). Motor scores (Unified Parkinson's Disease Rating Scale) did not correlate with a loss of connection strength (F = 0.02, df = 1, *p* > 0.1).

## 4 Discussion

We have shown that the regional expression of the *MAPT* gene in health is associated with regions of high connectivity, which predict the functionally relevant loss of connectivity in PSP, a neurodegenerative diseases associated strongly with progressive misfolding and aggregation of the protein tau. Similar changes were observed in PD, which is more associated with alpha-synculein pathology but for which the risk of disease and accompanying dementia are associated with tau haplotypes.

Accumulating evidence points to the susceptibility of highly connected hub regions to change in a wide range of neurological and neuropsychiatric disease (Crossley et al., 2014). This evidence includes neurodegenerative syndromes such as Alzheimer's disease, where the hub selectivity of connection strength has been linked to greater metabolic demands (Buckner et al., 2009). Highly connected brain regions can also be considered “expensive” to maintain because they have large numbers of connections, which may make them vulnerable to disease processes because of their high metabolism (Achard and Bullmore, 2007), or through “hub overload” of information that would usually be managed by other disease-affected brain regions (Stam, 2014). Furthermore, greater connectivity may increase the probability of transferring and receiving harmful protein species (Clavaguera et al., 2013, Liu et al., 2012).

Our findings extend these network-based hypotheses by linking global network properties directly to the underlying expression of native tau in humans. Global network properties have been found to mirror patterns in gene expression (Fulcher and Fornito, 2016, Rubinov et al., 2015, Vértes et al., 2016), and more specifically, the association between *MAPT* expression and connection strength reflects the importance of the tau protein in maintaining axonal integrity by stabilizing microtubules (Lindwall and Cole, 1984). In humans, previous imaging studies have linked single gene variants with patterns of change in brain imaging (Filippini et al., 2009, Mata et al., 2014, Whitwell et al., 2011). We go further to suggest that the regional variation in the *MAPT* gene expression in health can contribute to the selective vulnerability of a densely connected region specifically, more highly connected regions may be intrinsically susceptible to neurodegeneration mediated by tau dysfunction.

The correlation between *MAPT* expression and the proportional loss of connection strength was greater in PD than for PSP. The variability between these 2 diseases suggests that multiple genes and disease processes are involved in the loss of functional connections between brain regions. Our approach was to test an hypothesis based on a known gene associated with cognitive changes in the 2 disorders of interest. A more exploratory approach investigating all the available microarray data in the Allen brain atlas is technically possible (Hibar, 2015) and may identify other contributing genes but would require careful control of type I statistical error. Our study was not powered to conduct such a study.

In contrast to *MAPT*, we did not find a strong association between *SNCA* expression and functional connectivity in health, or loss of connectivity in disease. There was a weak association between 1 *SNCA* probe and connectivity in health, but this was not matched by changes in disease. This is despite the association of PD with alpha-synuclein pathology. Lewy bodies containing alpha-synuclein are the hallmark of PD pathology (Spillantini et al., 1997), and there is now emerging evidence that alpha-synuclein could spread across neural networks and cause aggregation and cell loss through a prion-like process (Hansen and Li, 2012, Li et al., 2008). There is a correlation between cortical deposition of alpha-synuclein and dementia (Hurtig et al., 2000); however, our cohort of PD patients specifically excluded patients with dementia. We suggest that the role of alpha-synuclein in PD without dementia is not relevant to the more cortically derived network measures that we employed to examine large scale network functional connectivity.

We have examined functional connection strength across the whole brain, but the brain's functional network can be examined as a series of macroscopic brain networks, some of which have been associated with individual neurodegenerative diseases (Seeley et al., 2009). In the context of our results, the recent finding that gene expression may predict connectivity patterns (Hawrylycz et al., 2015, Richiardi et al., 2015) raises the possibility that gene expression patterns and their products may contribute to the susceptibility of specific brain networks to specific neurodegenerative pathologies.

We identified a clinical effect of destabilizing highly connected hub regions by demonstrating that verbal fluency declines with a loss of connection strength. Verbal fluency is a key component of early cognitive impairment in both PD and PSP (Rittman et al., 2013) and arises from the orchestrated function of multiple brain regions (Henry and Crawford, 2004). Therefore, a loss of the coordinating function of highly connected brain regions is expected to have a detrimental effect on verbal fluency.

A strength of our study is the hypothesis-driven selection of genes relevant to PSP and PD. This contrasts with an exploratory data-driven approach (Hawrylycz et al., 2015, Richiardi et al., 2015), which may reduce the statistical power to detect relevant effects of individual genes. However, we recognize that there are likely to be other genes that play a role in the pathology underlying PD and PSP whose differential expression may contribute to pathogenesis.

There are a number of limitations to this study. First, the gene expression data and imaging data were necessarily obtained from different populations. Although our hypothesis was based on evidence for the involvement of tau in both PD and PSP, we recognize that gene-connectivity relationships may not exist in all disorders where there is a change in connectivity; and therefore, a more direct imaging-pathologic correlation in a single population would be the optimum approach. Second, the Allen database from which the gene expression data was derived includes only 6 subjects raising the possibility that interindividual variance may account for our findings. However, the main statistical comparison was among the 471 defined brain regions rather than the 6 individuals, and in 5 of the 6 subjects, we found similarly significant within-subject correlations between *MAPT* expression and connection strength demonstrating that our results were not dependent on 1 or 2 subjects. Finally, our results may not be specific for PD and PSP. It will be important to replicate our findings in independent cohorts and in other tau-associated neurodegenerative disorders such as Alzheimer's disease and frontotemporal dementia.

In conclusion, our results link molecular and imaging data in support of hypotheses of network vulnerability to neurodegenerative pathologies. We propose that brain regions expressing greater levels of the *MAPT* gene are more susceptible to neurodegeneration associated with the *MAPT* haplotype or mediated by tau pathology.

## Disclosure statement

Edward T. Bullmore is employed half-time by GlaxoSmithKline, holds shares in GlaxoSmithKline, and is a deputy editor of Biological Psychiatry. Boyd C.P. Ghosh has received honoria from UCB Pharm and GlaxoSmithKline.

## Figures and Tables

**Table 1:** Demographics for all patient groups

Demographic	Control	PD	PSP	*p*-value
Mean age (range)	66.7 (40.6–84.4)	66.8 (46.9–77.0)	70.8 (58.3–92.5)	ns
Handedness (R/L)	52/1	29/1	40/5	ns
Gender (M:F)	30/23	15/15	23/22	ns
UPDRS III (range)	—	20.8 (7–38)	36.7 (8–69)	<0.00001
PSPRS	—	—	38.3 (13–79)	—
MMSE (range)	29 (26–30)	28.4 (23–30)	24.5 (7–30)	<0.00001

The mean and range are shown where appropriate. Statistical comparison was made using analysis of variance for age and MMSE, *t* test for UPDRS, and chi-square test for handedness and gender.

Key: F, female; M, male; MMSE, Mini-Mental State Examination; ns, nonsignificant *p* > 0.1; PSPRS, Progressive Supranuclear Palsy Rating Scale, UPDRS, Unified Parkinson's Disease Rating Scale.
