# Supplementary material for: Regional expression of the MAPT gene is associated with loss of hubs in brain networks and cognitive impairment in Parkinson disease and progressive supranuclear palsy
Source: Neurobiol Aging. 2016 Dec;48:153–60. doi: 10.1016/j.neurobiolaging.2016.09.001 (PMC5096886; doi:10.1016/j.neurobiolaging.2016.09.001)
Supplement: Supplementary Figs. 1 and 2 [file mmc1.doc]

**Supplementary data**


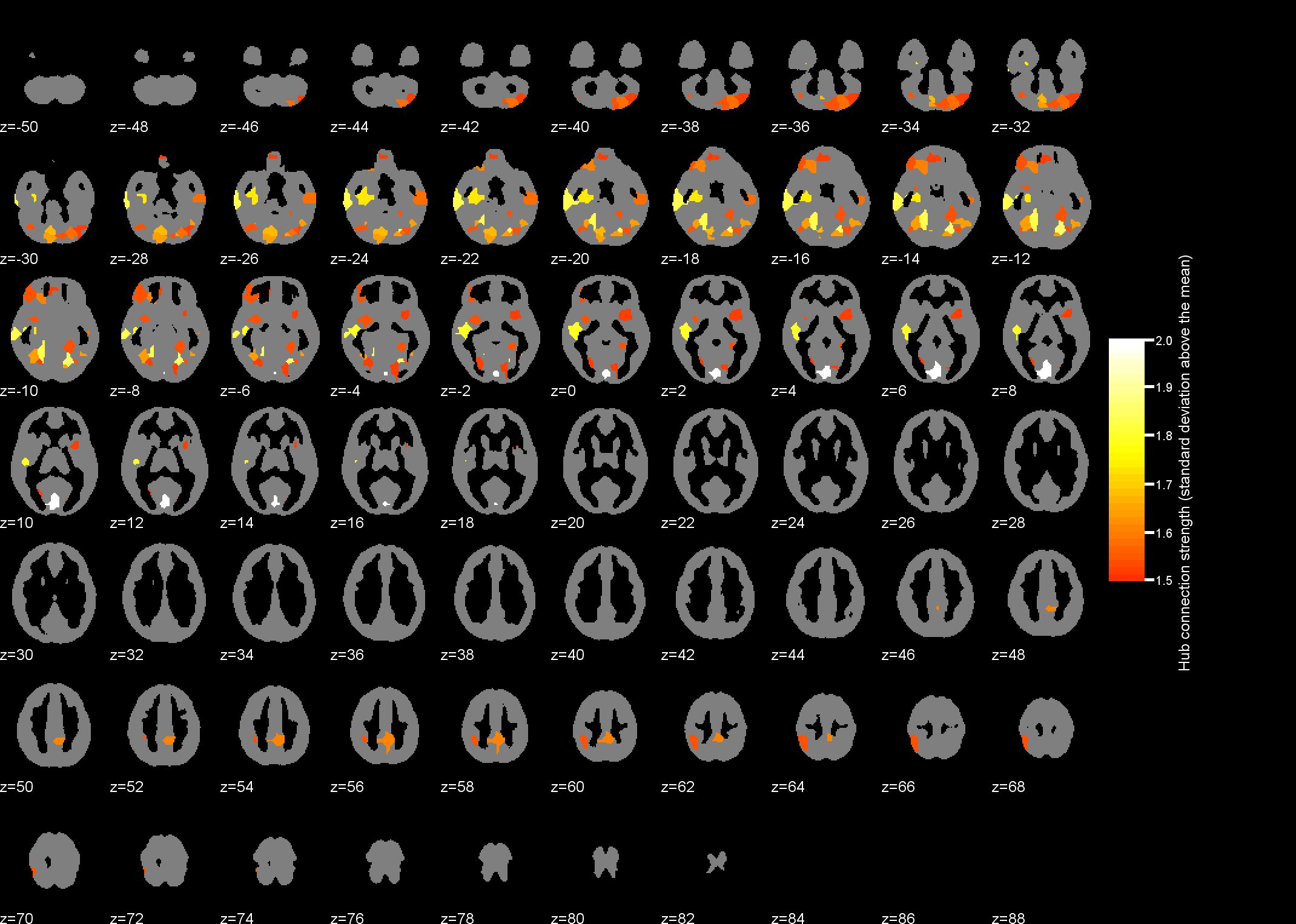
**Supplementary figure 1:** We approached the question of hubs from 2 perspectives: 1) binary hub vs non-hub, used in the correlation between hub connection strength and verbal fluency scores. To clarify the definition of hubs we now include a supplementary figure showing the location of brain regions defined as hubs ; 2) hub properties as a continuum of connectivity strength, and the continuous variable of connection strength (eg Figure 3). This figure demonstrates the hub regions that were defined using a binary approach. Using a threhold of 1.5 standard deviations above the mean, 38 hub regions were identified. The main regions identified as hubs were: precuneus, occipital cortex, occipital fusiform gyrus, right inferior frontal gyrus, bilateral temporal gyri, right superior temporal gyrus and right medial temporal lobe.


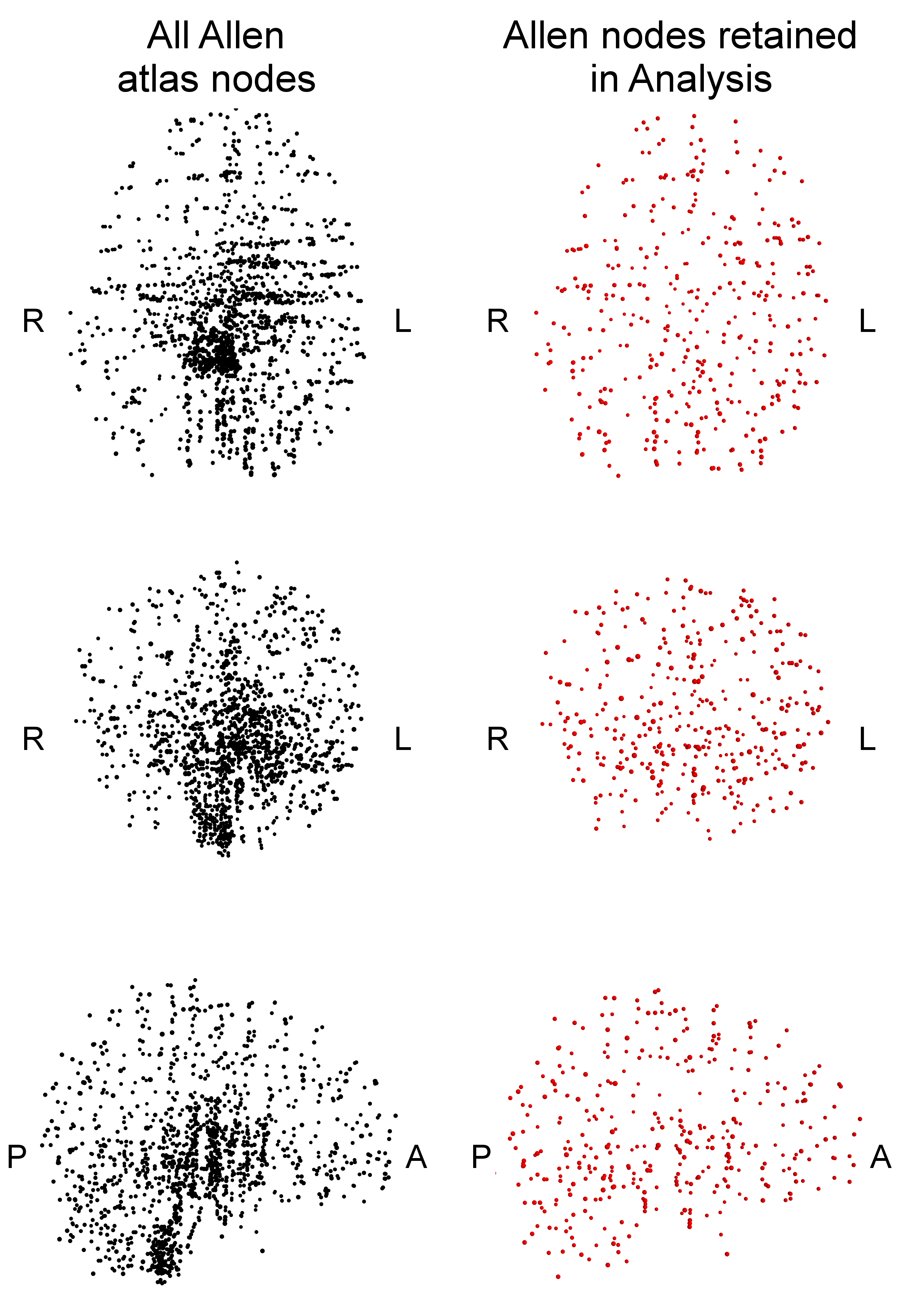


**Supplementary figure 2:** The Allen brain atlas is generated from 7 subjects with up to 1000 brain regions sampled. In order to match regions in the Allen atlas with brain imaging regions, a matching process was performed as described in the methods section. This figure demonstrates all the regions that were sampled in the original Allen brain atlas data on the left, and those regions retained after matching with imaging data on the right. Most cortical regions were maintained after matching, whereas the sampling from basal ganglia regions becomes more sparse after matching, and the brainstem was not included in the final analysis.
